# Association Between *GJA1* rs13216675 T>C Polymorphism and Risk of Atrial Fibrillation: A Systematic Review and Meta-Analysis

**DOI:** 10.3389/fcvm.2020.585268

**Published:** 2020-10-23

**Authors:** Xuejiao Chen, Guowei Li, Junguo Zhang, Xin Huang, Zebing Ye, Yahong Zhao

**Affiliations:** ^1^Center for Clinical Epidemiology and Methodology, Guangdong Second Provincial General Hospital, Guangzhou, China; ^2^Department of Health Research Methods, Evidence, and Impact, McMaster University, Hamilton, ON, Canada; ^3^Department of Cardiology, Guangdong Second Provincial General Hospital, Guangzhou, China

**Keywords:** atrial fibrillation, rs13216675, single-nucleotide polymorphism, systematic review, meta-analysis

## Abstract

**Background:** Rs13216675 T>C polymorphism, an SNP (single-nucleotide polymorphism) close to the gap junction protein alpha 1 (*GJA1*) gene, has been reported to be associated with risk of atrial fibrillation (AF); however, the results remained inconclusive. We aimed to perform a systematic review to clarify the relationship between rs13216675 and risk of AF.

**Materials and methods:** We systematically searched the databases of PubMed, EMBASE, Web of Science, and the Chinese National Knowledge Infrastructure up to July 15, 2020. Data were synthesized using the random-effects model. Odds ratios (ORs) and 95% confidence intervals (CIs) were calculated to estimate the relationship between rs13216675 and risk of AF.

**Results:** Seven studies involving 39,827 cases and 458,466 controls were analyzed in the meta-analysis. The overall pooled OR of rs13216675 polymorphism for AF was significant (OR = 1.10, 95% CI = 1.07–1.12, *P* < 0.001) under the additive genetic model. Subgroup analyses revealed that rs13216675 polymorphism was significantly associated with AF in both Asians (OR = 1.12, 95% CI = 1.07–1.17, *P* < 0.001) and Europeans (OR = 1.09, 95% CI = 1.06–1.12, *P* < 0.001). When data were stratified by control sources, rs13216675 polymorphism was significantly related to AF in studies with both population-based controls (OR = 1.09, 95% CI = 1.07–1.12) and hospital-based controls (OR = 1.12, 95% CI = 1.07–1.17). No evidence of publication bias was detected.

**Conclusion:** Our meta-analysis suggested that rs13216675 was significantly related to risk of AF and, therefore, might serve as a potential biological marker of AF.

## Introduction

Atrial fibrillation (AF) is the most common type of cardiac arrhythmia, affecting over 33 million individuals worldwide, with a prevalence of 5.96 per 1,000 in men and 3.73 per 1,000 in women (1, 2). AF is associated with high rates of cardiovascular and cerebrovascular morbidity and mortality, primarily related to the associated risk of stroke (3, 4). Currently, the pathogenesis of AF remains unclear, and the effective management of AF is still lacking, while only a small percentage of patients could receive catheter radiofrequency ablation or heart surgery to control rhythm (5). The high prevalence and low treatment of AF result in a growing health care cost and public health burden (4). Thus, it is necessary to improve understanding of the pathogenesis for AF to aid in the improvement in clinical outcomes.

Most epidemiological evidence indicates that male sex, hypertension, obesity, overweight, underweight, and diabetes are established risk factors for AF (5, 6). However, AF remains largely unpredictable by aforementioned risk predictors, suggesting that genetic variants may contribute to the risk of AF. Genome-wide association studies (GWAS) have identified some loci associated with AF (7–9). Gap junction protein alpha 1 (GJA1) is an intercellular channel that connects the cytoplasm of neighboring cells, allowing for crosstalk, and regulating the direct intercellular exchange (10). The SNP (single-nucleotide polymorphism) rs13216675 is located on chromosome 6q22 close to the *GJA1* gene. One study reported that rs13216675 was associated with the susceptibility to AF (11), and such association has been replicated in several populations (12, 13). However, in spite of the fact that the sample size was relatively small, some studies found no significant association between rs13216675 and risk of AF (14, 15), making the relationship still inconclusive. Given more and more recent epidemiological reports published, in this study, we aimed to conduct a systematic review and meta-analysis to clarify the association between rs13216675 and risk of AF. Summative evidence from this systematic review may provide some insight into prevention, treatment, and management of AF.

## Materials and Methods

### Literature Search

The study was performed in compliance with the Preferred Reporting Items for Systematic Reviews and Meta-Analyses (PRISMA) statement (16). A systematic literature search was performed on the PubMed, EMBASE, Web of Science, and CNKI (China National Knowledge Infrastructure) up to July 15, 2020. The terms “GJA1 OR rs13216675 OR susceptibility loci OR genes” and “atrial fibrillation OR AF” were combined to search for potentially relevant articles. This study was conducted without restriction on language. Our searches were not limited by publication status or setting. We also manually researched the references from the included studies for potentially related reports. Two independent reviewers (XC and GL) searched the literature and screened the records retrieved.

### Inclusion Criteria

The eligibility criteria included: (1) prospective or retrospective study on relationship between rs13216675 and early onset of AF; (2) prospective or retrospective study reporting incident of recurrent AF in patients after catheter ablation; (3) data available for extraction.

Studies were excluded if one of the following criteria was fulfilled: (1) not relevant to rs13216675 polymorphism and AF; (2) case reports or case series; and (3) no information for data extraction. For duplicate reports, only the study with the largest sample size was included.

### Data Extraction

A standardized data collection form was used to obtain the information from each study: the surname of the first author, publication year, the country of origin, population, study design, sample sizes of cases and controls, number of genotypes, the adjusted relative risk (RR) or odds ratio (OR) of AF with its 95% confidence interval (CI), *P*-value of HWE (Hardy–Weinberg equilibrium), and genotyping methods. Two reviewers (XC and GL) independently performed the data extraction and solved any disagreement by discussion.

### Statistical Analysis

All statistical analyses were conducted with STATA version 12.0. We performed a meta-analysis of the included studies using a random-effects model. We pooled the point estimates from each study using the generic inverse-variance method of Der Simonian and Laird. The heterogeneity of effect size estimates across these studies was quantified using the *I*^2^ statistic. Subgroup analyses were carried out by populations (Asian vs. European) and sources of control (population-based vs. hospital-based). The funnel plot and Egger's test were applied to evaluate potential publication bias.

### Quality Assessment

The Newcastle–Ottawa scale (NOS) was employed to assess the quality of eligible studies from three aspects: (1) selection of cases and controls; (2) comparability between cases and controls; and (3) exposure in cases and controls. The NOS has a score ranging from zero to nine, and a higher score indicates higher study quality. Studies with a score of more than seven points were considered as of high quality (17).

### Sensitivity Analysis

Five predetermined sensitivity analyses were performed by: (1) excluding studies reporting RRs (rather than ORs), (2) excluding studies investigating AF recurrence after catheter ablation (rather than onset of AF), (3) excluding studies not with a case-control design, (4) applying a fixed-effects model, and (5) excluding studies not with a low-risk-of-bias. Moreover, we conducted a *post hoc* sensitivity analysis by omitting one study at a time and calculating pooled ORs for the remaining studies.

## Results

### Characteristics of Included Studies

**Figure 1:**
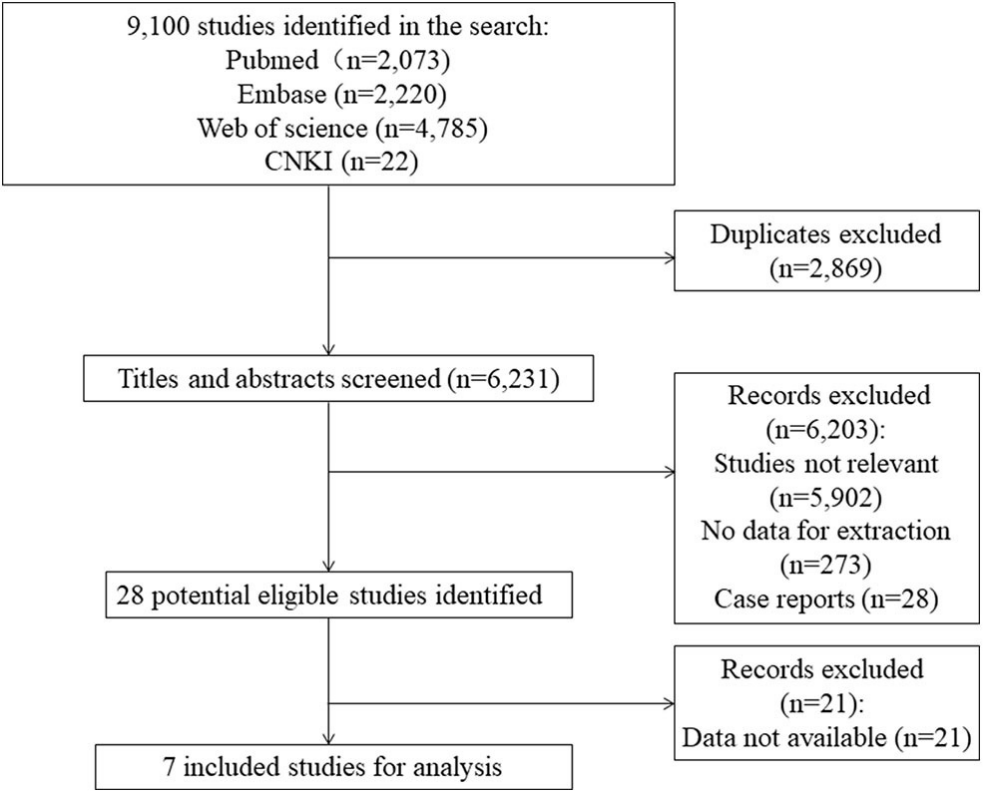
Flow diagram of study selection.

The literature search identified 9,100 studies from the databases. After excluding irrelevant and duplicate records by screening titles and abstracts, 28 potential eligible studies remained. We excluded 21 articles that had no data available for extraction. A total of seven studies containing 39,827 cases and 458,466 controls were included for analyses (Figure 1). Characteristics of the included studies are summarized in Table 1. There are 12,174 AF cases and 13,957 controls in Asians and 27,653 cases and 444,509 controls in Europeans. The mean ages varied from 50.0 (14) to 72.1 (13) years for AF cases, and from 52.4 (11) to 66.1 (11) years for controls, respectively. Five included studies satisfied the HWE hypothesis. The NOS scores of the included studies ranged from 6 to 8, indicating that the included studies were of relatively good study quality (Supplementary Table 1).

**Table 1:** Characteristics of included studies in the systematic review and meta-analysis.

**References**	**Population**	**Sample size (case/control)**	**Source of control**	**Hardy–Weinberg equilibrium (HWE)**	**Odds ratio/relative ratio [95% confidence interval (CI)]**	**Outcome measurement**	**Study design**	**Methods**	**NOS score**
Sinner et al. (11)	Europeans	13,398/69,570	Population based	Yes	1.10 (1.06–1.14)	Prevalent AF (early-onset AF)	Case-control study	Directly genotyped with the iPlex matrix-assisted laser desorption/ionization time-of-flight mass spectrometry technique	8
Sinner et al. (11)	Japanese	8,220/4,657	Population based	Yes	1.11 (1.05–1.17)	Prevalent AF (early-onset AF)	Case-control study	The multiplex polymerase chain reaction-based Invader Assay (Third Wave Technologies)	8
Wei et al. (15)	Chinese	600/600	Hospital based	No	1.111 (0.943–1.299)	Prevalent AF (postoperative atrial fibrillation)	Nested case-control study	A polymerase chain reaction-restriction fragment length polymorphism method (PCR-RFLP)	8
Zhao, 2016^*^	Chinese	1,150/1,150	Population based	Yes	1.16 (1.02–1.31)	Prevalent AF	Case-control study	iLMDR genotyping method	8
Lee et al. (14)	Koreans	872/5,512	Population based	Yes	1.10 (0.97–1.26)	Prevalent AF (early-onset AF)	Case-control study	An Affymetrix Genome-Wide Human SNP Array 6.0 chip	8
Thorolfsdottir et al. (13)	Icelanders	14,255/374,939	Population based	NA	1.08 (1.04–1.11)	Prevalent AF	Case-control study	Illumina SNP chips	7
Wang et al. (12)	Chinese	1,164/1,460	Population based	Yes	1.19 (1.04–1.35)	Prevalent AF	Case-control study	The high-resolution melting (HRM) analysis	7
Choe et al. (18)	Koreans	168/578	Hospital based	Yes	1.010 (0.782–1.305)	AF recurrence after ablation	Case-cohort study	TaqMan SNP genotyping assays	6

*NOS, Newcastle–Ottawa scale; HWE, Hardy–Weinberg equilibrium*.

* *Data not yet published [Zhao L. Genetic Variants in NEURL and PIIx2 are associated with the risk of atrial fibrillation. Nanjing Medical University (2016)]*.

### Overall and Subgroup Analyses

**Figure 2:**
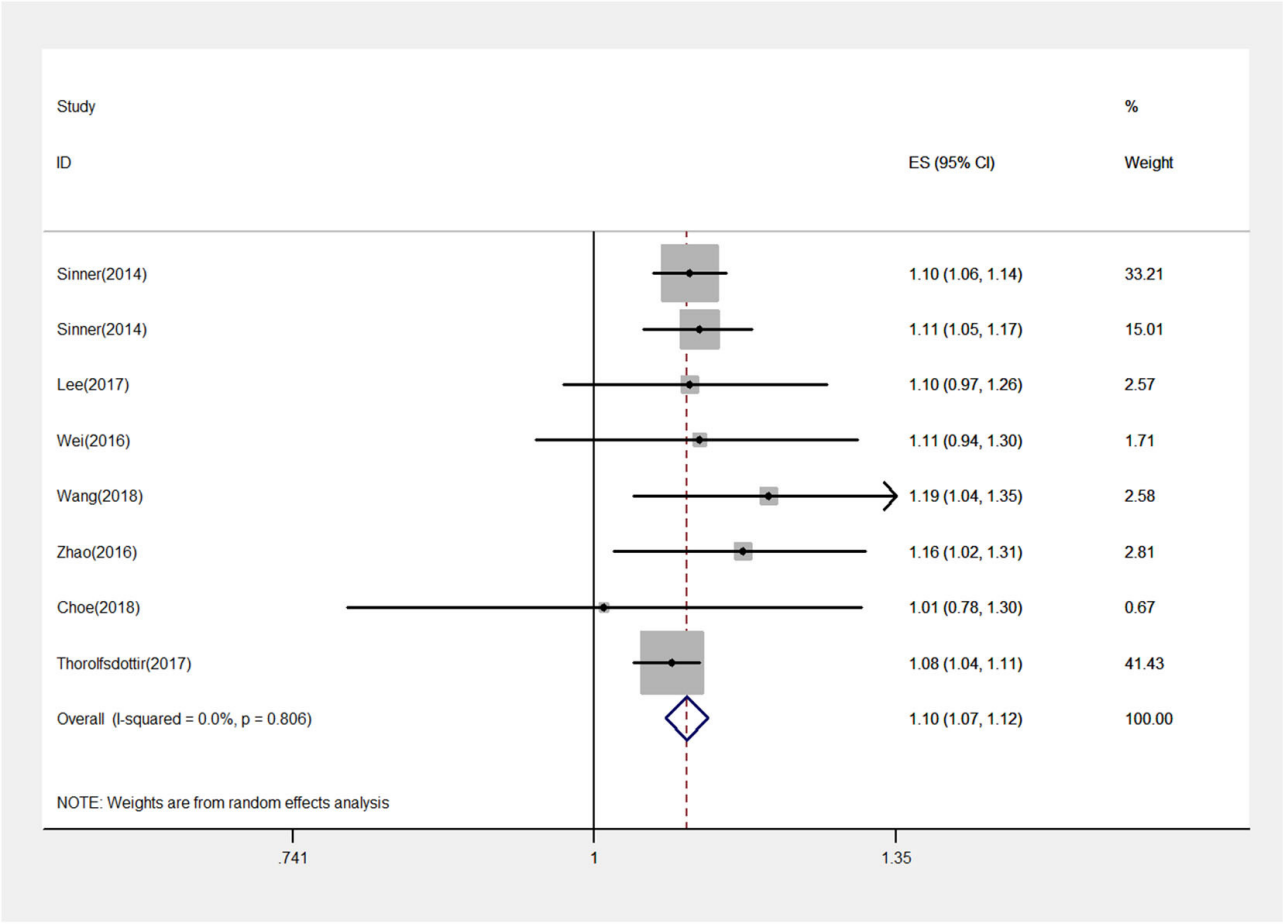
Forest plot for the rs13216675 and risk of atrial fibrillation (AF) using an additive model. The squares and horizontal lines correspond to the study-specific odds ratio (OR) and 95% confidence interval (CI), respectively. The area of the squares reflects the weight (inverse of the variance). The diamond represents the OR and 95% CI of the pooled results.

A total of 498,293 participants were analyzed. We found that rs13216675 was significantly associated with AF in the overall population under additive genetic model (OR = 1.10, 95% CI = 1.07–1.12, *P* < 0.001) (Figure 2). Similarly, rs13216675 polymorphism was significantly related to AF in both Asians (OR = 1.12, 95% CI = 1.07–1.17, *P* < 0.001) and Europeans (OR = 1.09, 95% CI = 1.06–1.12, *P* < 0.001). Moreover, a significant association between rs13216675 and risk of AF was observed in both studies with population-based controls (OR = 1.09, 95% CI = 1.07–1.12, *P* < 0.001) and hospital-based controls (OR = 1.12, 95% CI = 1.07–1.17, *P* < 0.001) (Table 2).

**Table 2:** Subgroup analysis of the association between rs13216675 and risk of AF.

**Group analysis**	**Number of studies**	**Atrial fibrillation (AF) size/control size**	**Pooled OR (95% CI)**	***P***	***I*^**2**^ (%)**
Total	8	39,827/458,466	1.10 (1.07–1.12)	<0.001	0
**Ethnicity**					
Asian	6	12,174/13,957	1.12 (1.07–1.17)	<0.001	0
European	2	27,653/444,509	1.09 (1.06–1.12)	<0.001	0
**Source of control**					
Hospital-based	4	10,152/7,295	1.12 (1.07–1.17)	<0.001	0
Population-based	4	29,675/451,171	1.09 (1.07–1.12)	<0.001	0

*CI, confidence interval; OR, odds ratio; AF size, the total number of AF cases; control size*.

### Sensitivity Analysis

**Figure 3:**
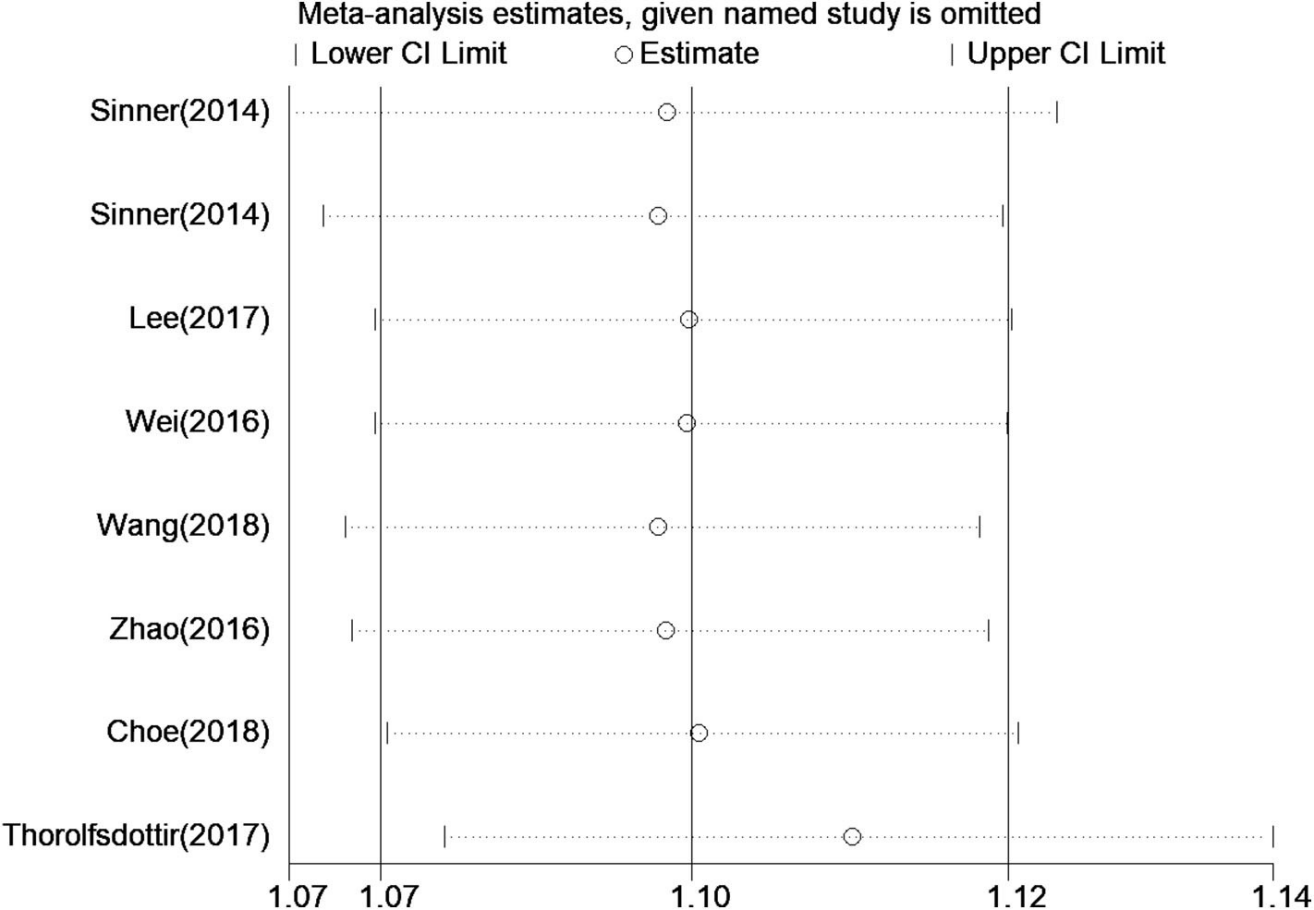
Sensitive analysis of the relationship between rs13216675 and risk of AF illustrating the influence of each study on pooled OR under the additive model. Results were computed by omitting each study in turn. Meta-analysis random-effects estimates (exponential form) were used. The two ends of the dotted lines represent the 95% CI.

Five *a priori* sensitivity analyses were conducted, with all reporting a statistically significant relationship between rs13216675 and risk of AF (Table 3). As shown in Figure 3, it was found that none of the individual studies substantially affected the overall result, with the ORs ranging from 1.09 (95% CI = 1.07–1.12) to 1.11 (95% CI = 1.08–1.14) for the association between rs13216675 and risk of AF, suggesting the robustness and insensitiveness of the overall result.

**Table 3:** Sensitivity analysis for the relationship between rs13216675 and risk of AF.

**Sensitivity analysis**	**Number of studies**	**AF size/control size**	**Pooled OR (95% CI)**	***P***	***I*^**2**^ (%)**
Excluding studies reporting RRs (rather than ORs)	6	18,209/384,239	1.09 (1.06–1.12)	<0.001	0
Excluding studies investigating recurrence of AF	7	39,659/457,888	1.10 (1.07–1.17)	<0.001	0
Excluding studies not with a case-control design	7	39,659/457,888	1.10 (1.07–1.17)	<0.001	0
Only including low-risk-of-bias studies	5	24,240/81,489	1.11 (1.08–1.14)	<0.001	0
Using fixed-effects model	8	39,827/458,466	1.10 (1.07–1.12)	<0.001	0

### Publication Biases

**Figure 4:**
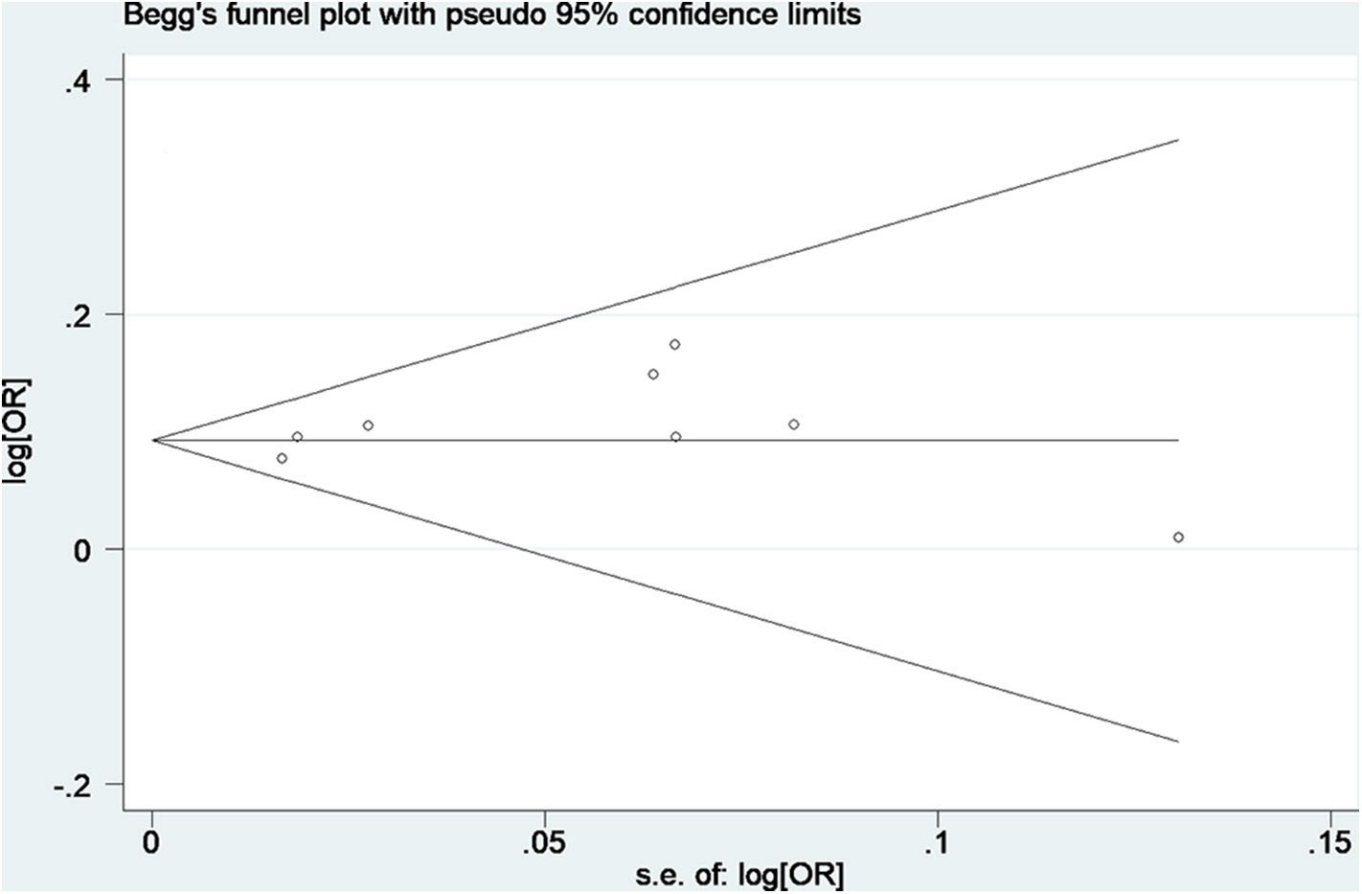
Begg's funnel plot of data from studies on the association of AF with rs13216675. Each point represents a separate study for the indicated association.

No obvious asymmetry of funnel plots was observed, indicating no evidence of potential publication bias (Figure 4). The Egger's test also found no significant publication bias, with a *P*-value of 0.28.

## Discussion

Our systematic review and meta-analysis aimed to ascertain whether rs13216675 T>C polymorphism was significantly associated with risk of AF. A significant relationship was found between rs13216675 and risk of AF (OR = 1.10, 95% CI = 1.07–1.12, *P* < 0.001) based on a total of seven studies. Findings from subgroup and sensitivity analyses supported the robustness of our main result.

Accumulating evidence indicates the *GJA1* gene as an important factor for AF susceptibility in the literature. The *GJA1* gene locates on chromosome 6q22.31 where it is a well-accepted AF locus (11). Connexin43 (Cx43), the cardiac gap junction protein encoded by *GJA1* gene, is abundantly expressed in the atrial and ventricular myocardium and the rapid ventricular conduction tissues (19). It was reported that the stress kinase c-jun N-terminal kinase (JNK) activation was found to be linked to the loss of gap junction Cx43, which impaired cell–cell communication between atrial myocytes and ultimately promoted AF development (20, 21). Therefore, it was suggested that a low expression of Cx43 was involved in AF through intercellular channel conduction. Intervention of Cx43 expression might be a novel therapeutic approach to AF (22). Although there is an advance in the emerging research of the *GJA1* gene and Cx43, evidence of rs13216675 (as an SNP close to the *GJA1* gene) for the potential mechanism related to AF susceptibility remains sparse and limited, leaving an important gap for further understanding of AF. In our systematic search, there was no study providing how rs13216675 may play a role in the development and progress of AF in detail. Only one recent report found that rs13216675 may play an important role in DNA methylation close to the *PKIB* gene CpG site to regulate gene expression and downstream biological processes by decreasing the methylation level, based on the data from Framingham Heart Study (23).

Sinner et al. first reported the association between *GJA1* rs13216675 and AF (11). Subsequently, Paludan-Muller et al. focused on this SNP and emphasized the importance of rs13216675 for AF susceptibility (24). As shown in Table 2, interestingly, we found no significant subgroup difference between Asian (OR = 1.12) and European (OR = 1.09) populations: *P* = 0.31 for subgroup difference. This may indicate a potentially consistent effect of rs13216675 on AF susceptibility across various ethnicities. However, as mentioned above, the pathophysiological mechanisms responsible for AF were still far from completely understood (24, 25). Given more and more published studies with inconclusive results, our current study may provide some insight into further clarification of the relationship between rs13216675 and risk of AF and may direct future research to potential targets of prevention, treatment, and management of AF.

To the best of our knowledge, this is the first systematic review and meta-analysis assessing the association between rs13216675 T>C polymorphism and risk of AF. We performed a comprehensive and exhaustive search to retrieve all relevant studies. Data were extracted and managed in duplicate with a good level of consensus between the independent reviewers. Robustness was supported by rigorous statistical analyses including subgroup and sensitivity analyses. It should also be noted that some limitations existed for our study. First, the association between rs13216675 and risk of AF may be influenced by gene–gene and gene–environmental interactions (26); however, we could not further explore such interactions due to limited data available for extraction. Although the included studies were considered as of relatively good quality (Table 1), there was only one study assessing the relationship between rs13216675 and risk of AF recurrence (18). Therefore, it remained largely unknown whether the association was similar to other populations and settings regarding AF recurrence after catheter ablation. One study did not provide data on the HWE test for the control group (13), and another study specifically did not satisfy the HWE hypothesis (15). Even though our *post hoc* sensitivity analysis showed that these two individual studies did not substantially influence our main result (Figure 3), it would require further investigation to assess how the lack of HWE test would impact the relationship between rs13216675 and risk of AF. Finally, we focused on one single genotype. In fact, both polygenic and monogenic factors contribute to AF risk in the general population, and the polygenic risk for AF explains a larger proportion of genetic susceptibility. We did not analyze the interactive effect between monogenic and polygenic risks (27). Taken together, given the limited data available in the literature, further studies are warranted to provide more detailed data on gene–environmental interactions and the generalizability of findings from our current study.

## Conclusions

In summary, our study found that rs13216675 polymorphism was significantly related to risk of AF and, therefore, might serve as a potential biological marker of AF. Further well-designed and adequately powered studies are needed to explore gene–environmental interactions and the potential targets related to rs13216675 for the prevention, treatment, and management of AF.

## Data Availability Statement

The original contributions generated for the study are included in the article/Supplementary Material, further inquiries can be directed to the corresponding author/s.

## Author Contributions

XC and GL were responsible for the study conception, design of the study, data acquisition, and analysis and interpretation of results. ZY and YZ were responsible for data acquisition. XC wrote the manuscript that was reviewed and revised by GL, JZ, and XH. All authors contributed to the article and approved the submitted version.

## Conflict of Interest

The authors declare that the research was conducted in the absence of any commercial or financial relationships that could be construed as a potential conflict of interest.

## Abbreviations

SNP: single-nucleotide polymorphism
GJA1: gap junction protein alpha 1
AF: atrial fibrillation
OR: odds ratio
CI: confidence interval.
