# Supplementary material for: Association Between GJA1 rs13216675 T>C Polymorphism and Risk of Atrial Fibrillation: A Systematic Review and Meta-Analysis
Source: Front Cardiovasc Med. 2020 Oct 23;7:585268. doi: 10.3389/fcvm.2020.585268 (PMC7649778; doi:10.3389/fcvm.2020.585268)
Supplement: Supplementary file 1 [file Table_1.DOC]

**Supplement Table 1**. Detailed scores of Newcastle-Ottawa Scale for each included study

| **Study by first author** | **Selection** | **Comparability** | **Exposure** |
| --- | --- | --- | --- |
| Sinner *et al* | 4 | 2 | 2 |
| Lee *et al* | 4 | 2 | 2 |
| Wei *et al* | 4 | 2 | 2 |
| Wang *et al* | 3 | 2 | 2 |
| Zhao *et al* | 4 | 2 | 2 |
| Choe *et al* | 3 | 1 | 2 |
| Thorolfsdottir *et al* | 4 | 1 | 2 |

Note- Rating criteria of NOS included selection, comparability and exposure. Selection: 1, is the case definition adequate? (if some independent validation was required, one point); 2, representativeness of the cases (if yes, one point); 3, selection of controls (if they were from community controls, one point); 4, definition of controls (if they had no history and new occurrence, one point). Comparability: comparability of cases and controls on the basis of design or analysis: 1, ethnicity (if yes, one point); 2, age (if yes, one point). Exposure: 1, ascertainment of exposure (if in reliable method, one point); 2, same method of ascertainment for cases and controls (if yes, one point); 3, non-response rate (if they were the same between cases and controls, one point).
